# Associations between body dissatisfaction and self-reported anxiety and depression in otherwise healthy men: A systematic review and meta-analysis

**DOI:** 10.1371/journal.pone.0229268

**Published:** 2020-02-25

**Authors:** Mathew Barnes, Purva Abhyankar, Elena Dimova, Catherine Best

**Affiliations:** Faculty of Health Sciences and Sport, University of Stirling, Stirling, Scotland; Northumbria University Faculty of Health and Life Sciences, UNITED KINGDOM

## Abstract

**Introduction:**

It is unknown whether male body dissatisfaction is related to anxiety and depression. This study investigates whether there is an association between body dissatisfaction and self-reported anxiety and/or depression in otherwise healthy adult males.

**Method:**

A systematic review was conducted using Preferred Reporting Items for Systematic Reviews and Meta Analyses as the reporting guideline. Four databases including CINAHL complete, Health Source: Nursing/Academic Edition, MEDLINE and PsycINFO were searched for observational studies with a correlational design. Studies were appraised using the Appraisal tool for Cross-Sectional Studies to measure quality and risk of bias. Data were extracted from studies to analyse and synthesise findings using content analysis and random effects meta-analyses in male body dissatisfaction and anxiety, depression, and both anxiety and depression.

**Results:**

Twenty-three cross-sectional studies were included in the review. Nineteen studies found positive correlations between male body dissatisfaction and anxiety and/or depression. Meta-analyses of Pearson’s correlation coefficients found statistically significant associations with body satisfaction for anxiety 0.40 (95% CI 0.28 to 0.51) depression 0.34 (95% CI 0.22 to 0.45) and both anxiety and depression outcomes 0.47 (95% CI 0.33 to 0.59). The quality appraisal found study samples were homogeneous being mostly ascertained through academic institutions where participants were predominantly young, Caucasian and with relatively high educational attainment. Measures of body satisfaction focused predominantly on muscularity and thinness.

**Discussion:**

This study provides the first pooled estimates of the correlation between body dissatisfaction and anxiety and depression in men. Findings need to be interpreted with respect to the samples and outcomes of the included studies. It is recommended that future research should increase the diversity of men in studies. Studies should measure a wider range of body dissatisfaction types found in men.

**Conclusion:**

The findings demonstrate that an association between male body dissatisfaction and anxiety and depression is likely to exist. Future research should address the temporal relationship between body dissatisfaction and anxiety and depression.

## Introduction

Body dissatisfaction is the negative perception of one’s own physical appearance [1]. It often develops during adolescence and can be triggered by experience of bullying, one’s perception of others’ expectations and media influence [2]. Body dissatisfaction can lead to health risk behaviours including disordered eating, excessive exercise, taking diet pills and steroid use [3], and is also associated with poor mental health including low mood, low self-esteem and psychiatric illnesses including eating disorders [1] and body dysmorphia [4]. It is, therefore, linked with numerous negative consequences. Body dissatisfaction has, also, been associated with self-reported anxiety and depression [1; 5], and consequently, it is plausible that anxiety and depression exacerbates the negative consequences of body dissatisfaction.

Research in body dissatisfaction has predominantly focused on women [6; 7], and has provided robust evidence that there is a relationship between female body dissatisfaction and anxiety and depression [8; 9; 10]. The importance of this subject, consequently, has received more attention in the media from a female perspective, for example, principal fashion houses having banned size zero models to make a statement against unrealistic body expectations [11]. In comparison, the relationship between body dissatisfaction and anxiety and depression is less acknowledged in adult males. Despite this, there has been an increase in media portrayals of male models, actors and action figure toys who are mostly young, tall, and have muscular bodies [12]. These media portrayals erroneously suggest that these male characteristics described are the ideal norm. It is believed that men who most rigidly adhere to these portrayed ideal norms are more likely to experience body dissatisfaction than those who do not [13], therefore, highlighting the seriousness of this issue.

There are currently very few reviews available to demonstrate the existence of adult male body dissatisfaction. It was found in one review that an array of male sub-populations reported body dissatisfaction, including younger and older men, men with different sexualities, and men with different body shapes including muscular body builders, males considered a normal weight and overweight men [14]. In another review it was found that exposing similarly diverse groups of men to media sources featuring attractive and muscular males induced body dissatisfaction [15]. Despite the limited evidence, both reviews highlight that body dissatisfaction is an issue for men, though, whether male body dissatisfaction is related to anxiety and/or depression remains unknown. The aim of this review is, therefore, to explore whether there is an association between body dissatisfaction and self-reported anxiety and/or depression in otherwise healthy adult males.

## Methods

The present review was conducted using the Preferred Reporting Items for Systematic Reviews and Meta-Analyses (PRISMA) guidelines [16], available in Supplementary Material: S1 Table. The author roles were assigned at the beginning of the systematic review; the entire review was actively completed by one author (MB), though, close supervision and instruction were provided by two additional authors (PA; ED) during each step of the systematic review described below. A further author (CB) conducted the meta-analyses. All steps were planned and agreed by the team of authors prior to completion. This included deciding upon the eligibility criteria, search strategy, screening method and data extraction and appraisal tools used.

### Eligibility criteria

The eligibility criteria were established using the SPIDER (Sample; Phenomenon of Interest; Design; Evaluation; Research type) framework [17] as illustrated by Table 1. SPIDER was employed because the research aim is absent from interventions and comparators, as the purpose of this systematic review was to identify a correlation between male body dissatisfaction and anxiety and/or depression. This meant that prospective studies and other studies that assess causality were beyond the scope of this review.

**Table 1:** SPIDER criteria.

SPIDER Criteria	Included	Excluded	Rationale
**S**ample	-Adult males (≥18 years) in otherwise healthy populations -Can include studies investigating additional populations if male data are reported separately	All other populations	The research aim/question focuses on otherwise healthy adult males.
**P**henomenon of **I**nterest	Body Dissatisfaction	Absence of Body Dissatisfaction	The research aim/question focuses on body dissatisfaction.
**D**esign	Assessment of correlation/relationship between variables	Intervention with any assessment	Interventional studies may increase the risk of reporting bias [18] due to affecting how men responded.
**E**valuation	Self-reported Anxiety and/or Depression	Absence of self-reported Anxiety and Depression	The research aim/question focuses on anxiety and depression.
**R**esearch Type	Quantitative, including mixed-methods	Qualitative	The focus was on ascertaining the quantified relationship, both presence and strength between the variables.
**Other**	**Included**	**Excluded**	**Rationale**
Geographic	All	None	Studies conducted in all countries were considered to account for cultural differences.
Language	English	Non-English	Due to lack of access to translators only English studies were included.
Publication Age	2008 to 2018	Before 2008	Male body dissatisfaction is now more apparent due to media influence than before [2], and therefore, it was decided to focus on the previous decade.
Literature Type	Peer-Reviewed	Non Peer-Reviewed	Only peer reviewed literature was included to assure some degree of rigour and quality.
Data Source	Primary studies	All Other	Primary studies offer first-hand accounts involving the population and phenomenon of interest.
Accessibility	Full-text	Full-text unavailable	Full access to article was required for comprehensive data extraction and quality appraisal.

The decisions made on the eligibility criteria were discussed and agreed by the authors. To avoid using arbitrary rules in informing SPIDER, criteria were established broadly enough to account for all relevant literature. The inclusion criteria for outcomes were limited to self-reported measures of anxiety and depression. Whilst it is accepted that a wide scale of emotions could be associated with anxious and/or depressive feelings, the decision was made to implement this restriction because anxiety and depression form part of the Diagnostic and Statistical Manual of Mental Disorders [19] and, therefore, can be measured using validated tools.

Additional criteria were also considered beyond SPIDER. The publication age of articles was restricted to 2008–2018 to account for the previous ten years of work in this phenomenon. This was because there would appear to be a greater prominence of male body dissatisfaction than there has been before [2; 5; 12], making this review opportune at the time of completion.

### Information sources and search strategy

Four electronic databases including CINAHL complete, Health Source: Nursing/Academic Edition, MEDLINE and PsycINFO were independently searched. The search strategy combined index terms (MeSH/subject headings/thesaurus) and free-text keywords along with truncations, proximity searches and wild cards that were collectively agreed by the authors. Search terms for ‘adult’ were combined with terms for ‘body dissatisfaction’ and ‘anxiety’ and ‘depression’. Search terms used in the MEDLINE database are provided in S2 Table.

### Study selection

Search results were first exported to RefWorks for removal of duplicates before transferring to Microsoft Office Excel for screening. The titles and abstracts of literature results were initially screened to assess their eligibility. All studies that appeared to meet the inclusion criteria at this stage were kept for full-text screening. Whilst only one author was primarily responsible for conducting title, abstract and full text screening, any uncertainties around eligibility were discussed with a second author to confirm study inclusion or exclusion. Systematic reviews were identified in the search and studies extracted for inclusion in this review. The systematic review reference lists, however, did not yield any additional studies for inclusion.

The literature search produced 3,849 results: 145 in Health Source; 449 in CINAHL Complete; 1,542 in PsycINFO; and 1,713 in MEDLINE. After 930 duplicates were removed 2,919 search results remained. A further 2,711 search results were excluded following title and abstract screening, leaving 208 articles remaining for full-text screening. After full-text review another 185 articles were excluded for reasons that failed to meet the eligibility criteria. A total of twenty-three articles met all of the inclusion criteria and were eventually included in the systematic review. The literature screening and selection process using a PRISMA flow chart is illustrated in S1 Fig.

### Quality appraisal

The Appraisal tool for Cross-Sectional Studies (Axis tool) is a validated quality appraisal tool to assess the methodological quality and risk of bias of cross-sectional studies for systematic reviews using twenty criteria [20]. This includes assessment of study aims/objectives, clarity of target populations, sampling procedures, representability of target population, appropriateness of risk factor and outcome variables and the metrics used to measure these, and clarity of results and discussions.

The AXIS tool does not provide an aggregated score on quality, as certain unfulfilled criteria may compromise quality in a greater or lesser extent in different articles [20], and could not, therefore, equally contribute towards determining the overall methodological quality of all studies. The general and overall quality of an article is left at the discretion of the author to make rationale judgements.

Studies were not excluded on the basis of quality. The quality appraisal was used to highlight strengths and weaknesses in study design and their potential impact on strength of evidence.

### Data extraction

The authors agreed on data items to extract which appeared to have significance on the relationship between male body dissatisfaction and anxiety and depression. A data extraction sheet was developed and modified following a pilot run on a sample of studies (n = 5). The worksheet was finalised following extraction from a further two studies, which yielded no further amendments.

Data were extracted on the following items: study details (author and date, location, category of body dissatisfaction and aims and objectives); study methods (design, setting, population, sample size, measures, data collection tools, data analysis), and study findings (participant characteristics, effect sizes to confirm association between body dissatisfaction and anxiety and/or depression, moderating factors, and authors conclusions). A blank copy of the final data extraction worksheet used is available for review in S3 Table.

### Data analysis and synthesis–content analysis

Data were synthesised using content analysis, which summarises text parts into categories using replicable and systematic methods. This is achieved by coding literature into meanings that are shared and contrasted between studies [21]. Content analysis is often used in qualitative research to find trends in text, but is also applicable to quantitative research by seeking data patterns to form predictions, such as measuring whether similar or dissimilar populations shared the same experiences and perceptions [22]. The codes created were then assigned to categories. Additional codes were added to categories as more articles in the systematic review were read to ensure categories were exclusive and exhaustive. Each category was established by the shared association between codes. For example, the category ‘sexuality’ could be defined, and therefore coded in different ways: heterosexual/straight, homosexual/gay/sexual minority and unknown/unstated sexuality. Using the same codes and categories systematically throughout all articles meant that the content analysis was more reliable.

The content analysis was conducted using a priori coding, as a basic idea of what categories would be created had already been considered due to piloting the data extraction worksheet. The coding terms were supplemented until data were extracted from ten studies when the codes for each category were saturated. These categories were considered saturated when all authors agreed that there were no other possible codes, and that all existing codes were unambiguous.

In order to label data and perform frequency counts on the collective codes within each category, Statistical Package for the Social Sciences version twenty-three was employed to form a tally of results that could be compared across studies. This process provided an overview of studies meaning that study characteristics could be described. This also meant that it was easier to identify any differences between studies that assessed body dissatisfaction and anxiety, body dissatisfaction and depression or body dissatisfaction and both anxiety and depression, as well as any differences between studies that did and did not find positive associations. Content analysis, conclusively, enriched data analysis and synthesis by being an informative method that used validated and reliable processes.

### Data analysis and synthesis–meta-analysis

Meta-Analyses were conducted using Stata version fifteen on body dissatisfaction and anxiety, body dissatisfaction and depression, and body dissatisfaction and both anxiety and depression to summarise and integrate the results from individual studies to increase precision in determining the relationships between these variables in men. Conducting these three meta-analyses would help to identify whether anxiety or depression correlated more strongly with body dissatisfaction in men, or whether both combined had a stronger correlation than either anxiety or depression alone.

Effect size estimates from included studies were extracted and reviewed for suitability with a second author. Pearson’s Correlation Coefficients and Regression Coefficients were the most frequently reported effect measures. The Regression Coefficients reported were adjusted for different covariates across studies, which meant that they could not be combined to measure the effect size. Pearson’s Correlation Coefficients were, consequently, used for the meta-analyses. Meta-analyses were conducted using random effects due to the heterogeneity in study measurement tools. Summary results including means, standard deviations, odds ratios and relative risks can provide limited information to form comprehensive findings where these are sporadically reported across studies [23]. As this was the case here, summary results were not included in the meta-analyses.

The majority of metrics in body dissatisfaction included in the meta-analysis indicated that higher scores resulted in greater body dissatisfaction, whereas three studies [34; 35; 41] all contradicted this by using metrics that were positively coded, meaning that higher participant scores indicated higher body satisfaction. Because of this, the Pearson’s Correlation Coefficient sign was reversed for these studies to enable inclusion in the meta-analysis. The correlations and corresponding standard errors from each study were transformed into z-scores for analysis before performing the meta-analyses. All results were then transformed back to the original correlations from z-scores for ease of interpretation.

## Results

### Study characteristics

Table 2 summarises the characteristics of studies included in the systematic review using the content analysis.

**Table 2:** Data extraction study attributes.

*Article*	*Geographic including Country and Setting*	*Body Dissatisfaction Variable*	*Health Outcome/s*	*Population*	*Male Sample Size n = (% of total sample)*	*Ages*^1^	*Sample Characteristics including Ethnicity*, *Education and Sexuality*	*Relationship between Body Dissatisfaction and Anxiety*^*2*^	*Relationship between Body Dissatisfaction and Depression*^2^
[24]	USA, Academic Institution	Muscularity and thinness	Depression	Students	Unknown (part of a mixed sex sample)	M = 23, SD = 7.2	**Ethnicity** 28% Caucasian, rest unknown **Education** All studying at University. No further details given. **Sexuality** NR	NR	**Pearson's r =** 0.35 BD and Center for Epidemiologic Depression Scales
[25]	USA, Remote internet access	Adiposity and Weight	Anxiety, Depression, Self-Esteem, Disordered Eating	Non-descriptive Males	74 (50)	Not provided for males only.	**Ethnicity** 79.1% Caucasian, rest unknown **Education** NR **Sexuality** NR	**Pearson’s r =** 0.44 Modified Weight Bias Internalization Scale and 21-item Depression Anxiety Stress Scales **β =** 0.45 Independent Variables (BMI, Dislike Subscale of Anti-fat Attitudes Questionnaire, Gender, Modified Weight Bias Internalization Scale) Dependent Variable (21-item Depression Anxiety Stress Scales	**Pearson’s r =** 0.44 Modified Weight Bias Internalization Scale and 21-item Depression Anxiety Stress Scales **β =** 0.45 Independent Variables (BMI, Dislike Subscale of Anti-fat Attitudes Questionnaire, Gender, Modified Weight Bias Internalization Scale) Dependent Variable (21-item Depression Anxiety Stress Scales
[26]	Canada, Remote internet access	Muscularity and thinness	Anxiety, Depression	Homosexual/ Sexual Minority Males	389 (100)	≥18	**Ethnicity** 21.6% African American, 48.1% Asian, 24.2% Latino, rest unknown **Education** Almost half had undergraduate degrees or greater (% not specified). Rest unknown. **Sexuality** 100% homosexual/ sexual minority	**Pearson’s r =** 0.56 Male Body Attitude Scale: Total score and Social Appearance Anxiety Scale **β =** 0.18 Independent Variables (Step 1: Age, Ethnicity, Household Income; Step 2: Anxiety, Male Body Attitudes Scale, Marlowe-Crowne Social Desirability Scale) Dependent Variable (Social Appearance Anxiety Scale)	**Pearson’s r =** 0.56 Male Body Attitude Scale: Total score and Social Appearance Anxiety Scale **β =** 0.18 Independent Variables (Step 1: Age, Ethnicity, Household Income; Step 2: Anxiety, Male Body Attitudes Scale, Marlowe-Crowne Social Desirability Scale) Dependent Variable (Social Appearance Anxiety Scale)
[27]	Italy, Academic Institution	Muscularity and thinness	Anxiety, Disordered Eating	Non-descriptive Males	359 (100)	R = 18–30, M = 20.4, SD = 3.3	**Ethnicity** 100% Caucasian **Education** All studying at University. No further details given. **Sexuality** 89.8% heterosexual, rest unknown	**Pearson’s r =** 0.39 Body Dissatisfaction and Social Anxiety	NR
[28]	Italy, Academic Institution	Muscularity and thinness	Anxiety, Disordered Eating	Students	409 (100)	R = 18–31, M = 23.1, SD = 3.5	**Ethnicity** NR **Education** NR **Sexuality** 92% heterosexual, rest unknown	**Pearson’s r =** 0.28 Body Dissatisfaction and Social Anxiety	NR
[29]	USA, Academic Institution	Muscularity and thinness	Anxiety, Disordered Eating	Students	135 (28.6)	M = 20.5, SD = 3.2	**Ethnicity** 70.9% Caucasian, 9.1% Latino, 8.9% African American, 3.8% Asian, 3% Biracial, rest classified as 'other' **Education** All studying at University. No further details given. **Sexuality** 95.3% heterosexual, 4.7% homosexual/ sexual minority	**Pearson’s r =** 0.36 Strength of Stereotypic Views of Men-Body Image and Eating Disturbance and Physical Appearance State and Trait Anxiety Scale-Weight	NR
[30]	Italy, Academic Institution	Muscularity and thinness	Anxiety, Self-Esteem, Disordered Eating	Young Males	551 (100)	R = 18–28, M = 20.82, SD = 4.43	**Ethnicity** 95% Caucasian, 2% Latino, 1% African, rest biracial **Education** All studying at University. No further details given. **Sexuality** 93% heterosexual, rest unknown	**Pearson’s r =** 0.36 Body Dissatisfaction and Insecure-Anxious Attachment	NR
[31]	Hungary, Academic Institution	Muscularity and thinness	Anxiety, Self-Esteem	Young Males	239 (100)	R = 18–39, M = 20.3, SD = 2.78	**Ethnicity** NR **Education** All studying at University. No further details given. **Sexuality** NR	**Pearson’s r =** 0.36 Muscle Appearance Satisfaction Scale and State-Trait Anxiety Inventory	NR
[32]	Thailand, Academic Institution	Muscularity and thinness	Anxiety	Young Males	25 (31.3)	R = 18–25, M = 21, SD = 1.4	**Ethnicity** NR **Education** All studying at University. No further details given. **Sexuality** NR	**Pearson’s r =** 0.22 Body Image Dissatisfaction and Social Anxiety **β =** -0.05 Independent Variables (Body Image Dissatisfaction, Fear Of Negative Evaluation) Dependent Variable (Social Anxiety)	NR
[33]	USA, Academic Institution	Muscularity and thinness	Anxiety, Disordered Eating	Heterosexual and Homosexual/ Sexual Minority Males	78 (100)	M = 19.31, SD = 0.89	**Ethnicity** 71.8% Caucasian, 17.9% Latino, 7.7% African American, 2.6% Asian **Education** NR **Sexuality** 50% heterosexual, 50% homosexual/ sexual minority	**Pearson’s r =** 0.64 Eating Disorders Inventory-3: Drive for Thinness and Physical Appearance State and Trait Anxiety Scale	NR
[34]	Canada, Academic Institution	Muscularity and thinness	Anxiety	Students	125 (37.5)	R = 18–25	**Ethnicity** NR **Education** All studying at University. No further details given. **Sexuality** NR	**Pearson’s r = -**0.49 Self-Presentational Efficacy Expectancy and Social Physique Anxiety	NR
[35]	USA, Academic Institution	Muscularity and thinness	Anxiety	Students	137 (40.7)	Not provided for males only.	**Ethnicity** NR **Education** All studying at University. No further details given. **Sexuality** NR	**Pearson’s r =** -0.08 Muscular Strength and Social Physique Anxiety Scale	NR
[36]	USA, Academic Institution	Muscularity and thinness	Anxiety	Non-descriptive Males	111 (38.9)	Not provided for males only.	**Ethnicity** 79% Caucasian, 11.1% African American, 4.2% Latino, rest classified as 'other' **Education** All studying at University. No further details given. **Sexuality** NR	**β =** -0.86 Independent Variables (Drive For Muscularity x Drive For Thinness) Dependent Variable (Body Anxiety)	NR
[37]	Australia, Academic Institution	Muscularity and thinness	Anxiety	Males with different ethnicities	55 (23.6)	Not provided for males only.	**Ethnicity** Caucasian and Chinese as umbrella ethnicity term (% unknown) **Education** NR **Sexuality** NR	**Pearson’s r =** -0.35 (Caucasian) Body Areas Satisfaction Scale and Anxiety in both Males and Females. Gender significantly moderated the result **Pearson’s r =** -0.29 (Chinese) Body Areas Satisfaction Scale and Anxiety in both Males and Females. Gender significantly moderated the result	NR
[38]	Serbia, Unknown location/ platform	Adiposity and Weight	Anxiety, Depression	Non-descriptive Males	200 (27.4)	Not provided for males only.	**Ethnicity** NR **Education** 56.4% degree level, 23% current University students. Rest unknown. **Sexuality** NR	**Pearson’s r =** 0.38 Body Dissatisfaction and Emotional Distress **β =** 0.43 Independent Variables (Step 1: BMI, Body Dissatisfaction; Step 2: BMI x Body Dissatisfaction) Dependent Variable (Emotional Distress)	**Pearson’s r =** 0.38 Body Dissatisfaction and Emotional Distress **β =** 0.43 Independent Variables (Step 1: BMI, Body Dissatisfaction; Step 2: BMI x Body Dissatisfaction) Dependent Variable (Emotional Distress)
[39]	USA, Canada and Western Europe, Remote internet access	Genital self-image	Anxiety	Non-descriptive Males	620 (100)	R = 18–76, M = 32.4, SD = 10.5	**Ethnicity** 79.2% Caucasian, rest unknown **Education** 77.7% had undergraduate degrees or greater, rest unknown. **Sexuality** 60% homosexual/ sexual minority, 40% heterosexual	**Pearson’s r =** 0.64 Body Image and Social Appearance Anxiety	NR
[40]	UK, Academic Institution	Muscularity and thinness	Depression	Students	765 (20.6)	≥18	**Ethnicity** NR **Education** All studying at University. No further details given. **Sexuality** NR	NR	Correlates of four levels of body image concern with Depressive Symptoms **OR (no concern) =** 0.38 **OR (mild concern) =** 1.64 **OR (moderate concern) =** 2.78 **OR (marked concern) =** 10.62
[41]	USA, Academic Institution	Muscularity and thinness	Depression, Self-Esteem, Disordered Eating	Non-descriptive Males	114 (40.1)	Not provided for males only.	**Ethnicity** 43% Caucasian, 21% Asian, 17% African American, 9% Latino, rest classified as 'other' **Education** All studying at University. No further details given. **Sexuality** NR	NR	**Pearson’s r =** -0.43 Positive Body Image and Depression
[42]	Italy, Academic Institution	Muscularity and thinness	Depression, Disordered Eating	Heterosexual and Homosexual/ Sexual Minority Males	255 (100)	R = 19–25	**Ethnicity** NR **Education** NR **Sexuality** 50.9% heterosexual, 49.1% homosexual/ sexual minority	NR	**Pearson’s r =** 0.53 (Heterosexual Men) Body Shame and Depression **Pearson’s r =** 0.37 (Homosexual Men) Body Shame and Depression **β =** 0.49 (Heterosexual Males) Independent Variable (Body Shame) Dependent Variable (Depression) **β =** 0.32 (Homosexual Males) Independent Variable (Body Shame) Dependent Variable (Depression)
[43]	USA, Remote internet access	Adiposity and Weight	Depression, Disordered Eating	Homosexual/ Sexual Minority Males	228 (100)	M = 31.1, SD = 12.7	**Ethnicity** 76% Caucasian, 6% Latino, 6% Asian, 2% African American, <1% Middle Eastern, <1% Native American, rest biracial **Education** NR **Sexuality** 100% homosexual/ sexual minority	NR	**Pearson’s r =** 0.33 Body Fat Dissatisfaction and Depression **β =** 0.29 Independent Variables (Step 1: Muscle; Step 2: Muscle, Body Fat) Dependent Variable (Depression)
[44]	Australia and New Zealand, Academic Institution	Adiposity and Weight	Depression	Young Males	274 (25.3)	M = 21, SD = 3	**Ethnicity** NR **Education** All studying at University. No further details given. **Sexuality** NR	NR	**Pearson’s r =** 0.3 Feelings of Fatness and Depression
[45]	Mexico, Academic Institution	Adiposity and Weight	Depression	Young Males	1,736 (47.9)	R = 18–20	**Ethnicity** NR **Education** NR **Sexuality** NR	NR	**RRR =** 1.1 self-estimation of weight status and Depression
[46]	USA, Academic Institution	Body Tan	Depression	Young Males	107 (47.1)	Not provided for males only.	**Ethnicity** 43% Caucasian, 33% Asian, 16% African American, 2% Hispanic, rest classified as 'other' **Education** NR **Sexuality** NR	NR	**Pearson’s r =** 0.04 Appearance Orientation and Depression

NR = Not Reported.

^**1**^ = range (R =), minimum age (≥), mean (M =), standard deviation (SD =), where male age is not provided separately males were at least 18 years old.

^2^ = Pearson Correlation Coefficient (Pearson’s r =), Regression Coefficient (β =), Odds Ratio (OR =), Relative Risk Ratio (RRR =), body dissatisfaction (BD).

The studies varied geographically; US (United States) (n = 9) [24; 25; 29; 33; 35; 36; 41; 43; 46], Italy (n = 4) [27; 28; 30; 42], Canada (n = 2) [26; 34], Australia (n = 1) [37], Hungary (n = 1) [31], Mexico (n = 1) [45], Serbia (n = 1) [38], Thailand (n = 1) [32], UK (United Kingdom) (n = 1) [40] or in multiple countries (n = 2) including US, Canada and Western Europe [39], and Australia and New Zealand [44]. Studies did not, however, vary according to their setting as 18 were conducted in academic institutions including colleges and universities [24; 27; 28; 29; 30; 31; 32; 33; 34; 35; 36; 37; 40; 41; 42; 44; 45; 46]. Other studies were conducted online in a remote environment (n = 4) [25; 26; 39; 43], whilst one study’s article did not report its setting [38].

The studies varied in the aspect of body dissatisfaction that they assessed; muscularity and thinness (n = 16) [24; 26; 27; 28; 29; 30; 31; 32; 33; 34; 35; 36; 37; 40; 41; 42], adiposity and weight (n = 5) [25; 38; 43; 44; 45], body tan (n = 1) [46] and genital self-image (n = 1) [39]. Anxiety and depression were varyingly investigated throughout studies; only anxiety (n = 12) [25; 26; 27; 28; 29; 30; 31; 32; 33; 34; 35; 36; 37; 38; 39], only depression (n = 8) [24; 25; 26; 38; 40; 41; 42; 43; 44; 45; 46], and both anxiety and depression (n = 3) [25; 26; 38].

The populations that studies investigated included students (n = 6) [24; 28; 29; 34; 35; 40], young males (n = 6) [30; 31; 32; 44; 45; 46], heterosexual men compared to homosexual/sexual minority men (n = 2) [33; 42], homosexual/sexual minority males (n = 2) [26; 43] and males with different ethnicities (n = 1) [37]. The remaining six studies made no reference to any specific group and so appeared to target all otherwise healthy men, which were subsequently labelled as non-descriptive males [25; 27; 36; 38; 39; 41]. Because the present review is investigating whether there is an association between body dissatisfaction and self-reported anxiety and/or depression in otherwise healthy adult males, it was decided to retain studies with samples assigned to non-specific groups, as they still met the eligibility criteria.

The total number of participants included in this review is at least 6,896. One article [24] did not state the number of males in their mixed-gender sample despite findings being segregated by gender, and therefore, the actual number of males is unknown. More than half of the studies included both males and females (n = 14) [24; 25; 29; 32; 34; 35; 36; 37; 38; 40; 41; 44; 45; 46]. Men represented less than half of the total sample size in all of these studies, except for Pearl and Puhl [25] where males represented fifty percent of the total sample. The remaining studies consisted solely of male samples (n = 9) [26; 27; 28; 30; 31; 33; 39; 42; 43].

The youngest and oldest subjects reported in studies were 18 and 76 years old respectively, and 12 studies used samples with a mean age between 18 and 25 years old [20; 27; 28; 29; 30; 31; 32; 33; 34; 42; 44; 45]. Information on age was, otherwise, scant throughout studies.

Caucasians significantly represented the greatest proportion of males across studies. Out of those studies where data on ethnicity were available (n = 10) 1,940 out of 2,377 (81.6%) males were Caucasian.

Across studies the vast majority of males were in higher education and/or already had a degree or higher. Out of those studies where this information was reported for males (n = 13), 3,476 out of 3,655 (95.1%) subjects were studying and/or highly qualified.

A wide range of sexuality groups were investigated throughout studies; predominantly heterosexual (n = 5) [27; 28; 29; 30; 42], equally heterosexual and homosexual/sexual minority (n = 1) [33], predominantly homosexual/sexual minority (n = 1) [39] or exclusively homosexual/sexual minority (n = 2) [26; 43]. The remaining fourteen studies did not report on sexuality (n = 14) [24; 25; 31; 32; 34; 35; 36; 37; 38; 40; 41; 44; 45; 46].

### Quality appraisal findings

S4 Table summarises the methodological quality of studies.

All cross-sectional studies included in the present review (n = 23) reported coherent aims and objectives, and used appropriate study designs for their investigations. Studies were, however, subject to sampling bias, as just two studies justified their sample sizes [33; 42], and only three studies stated with clarity who their target populations were [33; 34; 42]. The sampling frames prescribed were only appropriate in nine studies [24; 25; 26; 28; 32; 34; 35; 39; 40]; often restricted to academic institutions despite students and the well-educated not being the only male groups of interest, meaning that when not investigating students, studies regularly failed to entirely represent their intended populations. Few studies reported appropriate sampling methods that were not based on convenience (n = 3) [26; 39; 40], which suggests that in the majority of studies only the most accessible men participated in studies, again, reducing any opportunity to be representative of intended populations.

All studies, conversely, reported appropriate risk and outcome variables, and used validated metrics to measure these. This demonstrates use of high quality data collection methods. The study results, however, were often at risk of estimation bias. This is because just eight studies clearly reported joint and extensive use of precision estimates including probability values, confidence intervals and/or odds ratios [25; 31; 32; 33; 39; 40; 43; 45], meaning that statistical significance measures were visibly employed in less than half of study analyses, resulting in reduced data assessment quality.

The study findings all provided adequate descriptions of data, yet, only five studies attempted to quantify the level of non-response [26; 28; 40; 45; 46], consequently, assessing whether findings were subject to non-response bias. Except for El Ansari *et al* [40], none of the studies attempted to compare their samples’ findings with any available data from non-respondents to increase population representativeness. The sampling choices made and limited effort undertaken to address non-response are likely to have meant that studies failed to reach all intended groups of men to represent the otherwise healthy adult male population. Most studies (n = 17), despite this, presented internally consistent findings by providing results for all included participants [26; 27; 28; 29; 30; 31; 32; 33; 34; 35; 37; 39; 40; 42; 43; 44; 45]. The analyses for all tests described in the methods of the studies were also documented in their findings, again suggesting high quality results sections in articles.

All studies provided conclusions that were justified by their findings and that matched the intentions of their stated aims/objectives, and all but one study reported their methodological limitations [32]. Only two studies, however, reported methods that meant their designs were repeatable [39; 40]; descriptions of methods used were often poorly reported or completely omitted, meaning that they were mostly conducted using non-transparent methods. It is unknown, consequently, entirely how these studies were conducted, meaning that the robustness of study findings were questionable.

Two studies failed to report whether they had been reviewed and approved by an ethics board [36; 38]. Only one study declared both funding sources and conflicts of interest throughout the duration of their investigations [44]. Whilst this does not necessarily mean that studies were funded by bodies with particular agendas or suffered from incompatible views of different researchers, this regardless does raise questions concerning any possible funding and publication bias.

### Analysis and synthesis of findings

#### Body dissatisfaction and anxiety

Out of twelve studies eight found positive correlations between body dissatisfaction and anxiety in otherwise healthy adult males. The correlational relationship between muscularity and thinness related male body dissatisfaction and anxiety was assessed in eleven studies, nine of which realised statistically significant positive correlations. All of these studies were conducted in academic institutions.

These include Dakanalis *et al* [27] who found that anxiety strengthened the symptomatology of pre-existing relationships between male body dissatisfaction and disordered eating. In another study, Dakanalis *et al* [28] identified that anxiety reinforced the link between body dissatisfaction and disordered eating in students. Tantleff-Dunn *et al* [29] found that anxiety and disordered eating, also, significantly correlated with body dissatisfaction in students. Additionally, Dakanalis *et al* [30] found that anxiety was linked with body dissatisfaction, disordered eating and low self-esteem in young males. And in a study by Czeglédi *et al* [31], anxiety correlated with body dissatisfaction in young males, and that having a greater dissatisfaction with one’s own muscularity led to reduced self-esteem, and subsequently, increased trait anxiety. In another study, Pawijit *et al* [32] found a link between body dissatisfaction and anxiety in students, which was increased by fear of negative evaluation. Further to this, Carper *et al* [33] identified that body dissatisfaction correlated with anxiety, which was magnified by pressures attributed to media influence. Whilst this was apparent amongst all males, this correlation was more significant in homosexual men than heterosexual men. Gammage *et al* [34], also, found that men who self-perceived their body image as unappealing to others had greater social physique anxiety. And finally, Chu *et al* [35] highlighted that a lack of muscularity and strength increased levels of anxiety.

Not all studies investigating muscularity and thinness related body dissatisfaction found positive correlations with anxiety, such as Kelley *et al* [36], where muscularity and thinness related body dissatisfaction did not relate to anxiety in men. In addition to this, Hui and Brown [37] explored the relationship between body dissatisfaction and anxiety in men with different ethnicities; a negative association existed between body dissatisfaction and anxiety in Caucasian and acculturated and non-acculturated Chinese males.

One last study assessed the relationship between genital self-image related body dissatisfaction and anxiety; Loehle *et al* [39] conducted an internet study to identify that social appearance anxiety significantly correlated with a higher genital self-image, resulting in men having a worse perception of their own bodies regardless of age or sexuality.

These findings suggest that body dissatisfied men are more likely to report anxiety, compared to men who are not body dissatisfied, though, the evidence is largely limited to muscularity and thinness related body dissatisfaction. Studies were predominantly conducted in westernised countries and in academic institutions, utilising samples who were mostly young and educated Caucasian men, though, studies did report findings based on a wide range of sexuality groups. Data on sample characteristics were, however, not always reported.

#### Body dissatisfaction and depression

The relationship between body dissatisfaction and depression in otherwise healthy adult males was investigated in eight studies; seven found positive correlations. The correlational relationship between muscularity and thinness related male body dissatisfaction and depression was investigated in four studies, which were all conducted in academic institutions. These include El Ansari *et al* [40], who identified that students dissatisfied with their bodies reported depressive feelings whereas non-dissatisfied students did not report any depression. Edman *et al* [24], similarly, found a positive correlation between body dissatisfaction and depression in students. In another study, Gillen [41] discovered that having a positive body image was related to lower levels of depression, disordered eating and drive for muscularity as well as higher self-esteem amongst men. Finally, Dakanalis *et al* [42] found that previous exposure to sexually objectifying media strengthened the correlation between both body dissatisfaction and depression in all men, but more so in homosexual than heterosexual men. Body dissatisfaction, also, correlated with disordered eating in homosexual men.

A further three studies investigated the association between adiposity and weight related body dissatisfaction and depression; Blashill [43] found that body dissatisfaction correlated with depression and disordered eating in homosexual/sexual minority males in an online study. Two further studies were conducted in academic institutions, including Mulgrew *et al* [44], who identified a weak but positive correlation between body image perception and depression in young males who practiced unhealthy weight behaviours. Converse to Blashill [43] and Mulgrew *et al* [44], Andrade *et al* [45] failed to find any correlation between body dissatisfaction related to weight, BMI and depression in young males.

One final study conducted in an academic institution investigated the association between body tan related body dissatisfaction and depression [46]. A weak but positive correlation was found between young males who reported tanning indoors and greater depression than those who tanned outdoors, and that those with greater depression had body dissatisfaction.

The positive correlational relationship between body dissatisfaction and depression, similarly to anxiety, pertains to muscularity and thinness. Whilst no studies investigated male body dissatisfaction and anxiety alone in adiposity and weight, two studies did report positive correlations between adiposity and weight related body dissatisfaction and depression. All studies were conducted in western countries, and male subjects were, again, mostly young, educated and Caucasian. Studies predominantly took place within academic institutions. Data on sample characteristics were similarly unreported to investigations in anxiety. Sexuality was predominantly unreported in studies, but again was widely represented where data were available.

#### Body dissatisfaction and anxiety and depression

The association between male body dissatisfaction and both anxiety and depression was investigated in three studies only. These included Brdarić *et al* [38] who examined the moderating effect of adiposity and weight related body dissatisfaction on the relationship between weight and reported wellbeing in non-descriptive males. It was found that body dissatisfaction positively correlated with anxiety and depression. The study was conducted in an unknown setting. In a similar study conducted online, Pearl and Puhl [25] found that a moderate, positive correlation between body mass index (BMI) and anxiety, depression, disordered eating and low self-esteem existed, with the strongest correlation credited to those who were overweight or obese. In another study assessing muscularity and thinness related body dissatisfaction, Hart *et al* [26] found that anxiety significantly correlated with body dissatisfaction, drive for muscularity and disordered eating, and weaker but significant correlations with depression in homosexual/sexual minority men.

These studies therefore demonstrated positive associations between two types of body dissatisfaction and both anxiety and depression. The studies were all conducted in western countries, and either in remote or unknown location settings. The availability of data in ethnicity, education and sexuality were mixed. The methodological quality was similar across these studies. Additional research is required to confirm whether this relationship is valid due to the small number of studies available, and therefore whether it is applicable to large groups of men with different characteristics.

### Meta-analysis findings

Five studies did meet the eligibility criteria for the systematic review, but did not have data for inclusion in the present meta-analysis: Edman *et al* [24] used a mixed sex sample and did not clearly report the sample size of male participants; Kelley *et al* [36] did not calculate Pearson’s Correlation Coefficient using variables for ‘Drive for Muscularity’ and/or ‘Drive for Thinness’ and ‘Body Anxiety’; Hui and Brown [37] used a mixed sex sample and did not report separate results for males, despite gender having been reported to influence the relationship between variables; El Ansari *et al* [40] did not calculate Pearson’s Correlation Coefficient using variables for ‘Body Image Concern’ and ‘Depressive Symptoms’; and Andrade *et al* [45] did not calculate Pearson’s Correlation Coefficient using variables for ‘Estimation of Weight Status’ and ‘Depression’.

All other studies (n = 18) were included in the meta-analysis regardless of findings to reduce the risk of publication bias. Stratification was considered unsuitable for including the ineligible studies in a meta-analysis due to being unable to group them in a relevant way that reflected the systematic review’s aim.

S2 Fig shows a random-effects meta-analysis [47] conducted for body dissatisfaction and anxiety, which included ten studies [27; 28; 29; 30; 31; 32; 33; 34; 35; 39]. There was substantial heterogeneity in effects (Cochran’s Q = 102.00, p<0.001, I^2^ = 91.2%) between studies. The pooled correlation of effect was 0.40 (95% CI 0.28 to 0.51).

S3 Fig shows a random-effects meta-analysis [47] for body dissatisfaction and depression, which included five studies [41; 42; 43; 44; 46]. Because Dakanalis *et al* [42] assessed the correlation between body dissatisfaction and depression separately for heterosexual and homosexual men, the study was included two separate effect estimates in the meta-analysis. There was, again, substantial heterogeneity in the study effects (Cochran’s Q = 19.01 p<0.002 I^2^ = 73.7%).The pooled effect estimate was 0.34 (95% CI 0.22 to 0.45).

S4 Fig shows a random-effects meta-analysis [47] conducted for body dissatisfaction and both anxiety and depression, which included three studies [25; 26; 38]. Similarly to the other two analyses there was heterogeneity in effects (Cochran’s Q = 7.49, p = 0.024, I^2^ = 73.3%) between studies. The overall pooled correlation was 0.47 (95% CI 0.33 to 0.59).

Publication bias was assessed with Begg’s adjusted rank correlation test. This test assesses publication bias by determining if there is a significant correlation between the effect estimates and their variances [48]. For the anxiety meta-analysis the adjusted z score was 0.63, p = 0.53, for the depression meta-analysis the adjusted z score was 0.38, p = 0.70, and for the anxiety and depression meta-analysis the adjusted z score was -0.52, p = 0.60. These results indicate there is no evidence of publication bias.

## Discussion

The aim of this review was to investigate whether there is an association between body dissatisfaction and self-reported anxiety and/or depression in otherwise healthy adult males. A comprehensive search and selection strategy identified twenty-three studies that were described and synthesised following data extraction and analysis. Studies were quality appraised using the AXIS tool. Data were analysed and synthesised using content analysis and meta-analysis assessments. Nineteen studies found statistically significant positive correlations between body dissatisfaction and anxiety and/or depression. The results suggest that men who experience higher body dissatisfaction are more likely to also report higher levels of anxiety and depression.

It is undetermined whether anxiety or depression is more associated with body dissatisfaction, or whether they equally perpetuate this. There was significant levels of heterogeneity detected in each of the analyses conducted, as a result of inconsistency in the different effect sizes and confidence intervals demonstrated by each forest plot. This may be due to the different metrics used in individual studies, differences in body dissatisfaction perceptions between individuals and male populations, and/or the extent that body dissatisfaction causes concern for these participants, making the true effect different in studies. Heterogeneity may, also, be caused by another unknown reason.

The use of a random-effects model takes into account these differences suggested, assuming that the different effect sizes are not due to chance alone. However, in order for a random-effects model to be effective there must be a substantial number of studies included in the analysis, as results are to be generalised beyond that of the studies included in the analyses to the wider population [49]. Whilst the meta-analysis investigating both anxiety and depression did realise the highest effect score, this did include the smallest number of studies compared to both other meta-analyses. Because of this, and to ensure that heterogeneity is accounted for properly by a random-effects model, more research is needed to confirm if there is a distinction between anxiety and depression, and their influence on male body dissatisfaction.

Studies most commonly associate male body dissatisfaction with muscularity and thinness. This may just be a presumption made by the media, hence why it is investigated more. Other categories of body dissatisfaction may be equally as important in being associated with anxiety and depression such as adiposity and weight-related body dissatisfaction, which identified positive correlations with anxiety and depression and depression alone. How different types of body dissatisfaction affect anxiety and depression in men remains to be explored.

Future research should aspire to investigate these issues. This can be achieved by conducting further studies that assesses the association between body dissatisfaction and both anxiety and depression to detect if there are differences between these health outcomes. Studies should, also, measure the extent that different types of body dissatisfaction matter to men, thereby determining if they value the appearances of some body parts more than others. These assessments can be made by using additional metrics in the same investigations to produce more insightful findings.

Metrics in body dissatisfaction, however, tend to focus on adiposity and weight [50]. It is arguable that women, generally speaking, are more concerned with a drive for thinness [6; 7; 8; 9; 10] compared to men who desire more muscular bodies in modern society [13; 14; 15]. Additional metrics used in male body dissatisfaction should alternatively focus on muscularity and the areas of the body where this is deemed important such as the chest, shoulders and arms [13]. Using these metrics in combination with existing ones that focus on adiposity and weight as well as other body parts will provide an overall view of male body dissatisfaction and its primary indications, meaning that this can be better recognised in future sooner.

Having confirmed that there is a correlation between male body dissatisfaction and anxiety and depression in this systematic review, it would further validate the existence of this relationship if more could be understood between these variables. This, consequently, would add more robust evidence to this argument. One way of achieving this is longitudinal research, which is conducted over lengthy periods of time, and can provide a better insight into the order of variables and outcomes as they occur [51]. Metrics could, therefore, be given to subjects periodically to help determine when body dissatisfaction will likely exhibit anxiety and/or depression, or vice versa. Using longitudinal research in this way would increase rigour to this investigation whilst still conducting an observational study and maintaining a low reporting bias risk.

The majority of studies were conducted in academic institutions in westernised countries where mostly Caucasian young students and highly educated men were sampled. Because samples were predominantly from within these settings and populations this limits generalisability, and thus, similar studies should be conducted in more diverse research arenas.

The cultural differences between the geographic locations and ethnicity of men and experiences of acculturation could have implications for body dissatisfaction. This is because expectations and viewpoints in different population groups can vary. For example, Lau *et al* [52] found that both low and high acculturated Asian-American females experienced body dissatisfaction differently according to how acclimatised to western culture they were. It is, therefore, unknown whether these characteristics would be experienced similarly by males in these different population groups.

The collective findings do showcase diversity in sexuality, and have demonstrated that the relationship between male body dissatisfaction and anxiety and depression appears to be more prominent amongst homosexual than heterosexual men. But whereas McCabe and Ricciardelli [14] and this review both found that homosexual men were most susceptible to body dissatisfaction, these updated findings have confirmed that it is now becoming more apparent in heterosexual men too. This may be explained by more men becoming increasingly concerned with their physical appearance than ever before [2]. Future research should further explore the link between sexuality, body dissatisfaction and anxiety and depression.

The quality appraisal found similar strengths amongst all studies included in the systematic review. These included coherent aims/objectives, appropriate study designs, adequately described results for all tests stated in methods, few missing data, appropriate outcome measures, appropriate use of validated metrics to assess outcome measures, findings that were justified in the context of study aims/objectives, reported limitations and reported research ethics committee approval.

The quality appraisal equally found limitations that were shared across studies. Firstly, sample sizes were not justified and failed to determine the significance of their findings, and thus how valid they are. Most studies also failed to state target populations by neglecting to report their eligibility criteria. Furthermore, sampling frames and methods used meant that studies were restricted to the uniform sample characteristics described. The effects of these procedures increases the risk of selection bias, as well as non-response bias due to failing to identify non-western and non-Caucasian males outside of student environments.

Whilst probability values were widely reported, the majority of studies failed to report confidence intervals, though, the representativeness of sampling methods employed remains in question. Odds Ratio calculations were, also, seldom reported. It is therefore undetermined how strong the association between body dissatisfaction and anxiety and/or depression in otherwise healthy adult males is across studies, besides the combined meta-analysis results, meaning that findings are subject to estimation bias.

Finally, the quality appraisal identified that most studies did not report information on funding sources and/or conflicts of interest. It is, consequently, unknown whether findings presented are a true reflection of the association between body dissatisfaction and anxiety and/or depression, which unknowingly may mislead future health messages and interventions by presenting inaccurate findings.

The quality of cross-sectional research should also be considered. Whilst cross-sectional research is useful for conducting investigations at singular points in time to retrospectively assess the correlation between two or more investigative variables, confounders and their rate of exposure [18], it is methodologically flawed. This is because both independent and dependent variables are being investigated at once, meaning that causality cannot be determined [18]. Although beyond the scope of the present review, this meant that none of the studies were able to confirm whether body dissatisfaction causes anxiety and/or depression or vice versa.

As stated, the majority of studies have paid significant attention to the possible link between muscularity and thinness related body dissatisfaction and anxiety and depression. It is arguable that modern society wants men to believe that they should be more muscular. For example, television advertisements are increasingly portraying muscular adult males in a wide variety of scenarios unrelated to their physical appearance, health or beauty [12; 13; 15]. Consequently, men are being subjected to what media influence would determine to be the ideal norm for male body image. This may, therefore, exacerbate the negative consequences of body dissatisfaction for some men who are anxious and/or depressed, or may lead to anxiety and/or depression development.

These negative consequences could stem into numerous problems. Health risk behaviours such as disordered eating and excessive exercise that are associated with body dissatisfaction may become compulsive [3]. This persistent and increasing negative impact on mental health could continue to spiral into psychiatric illnesses, which could jeopardise physical health and wellbeing [1; 5]. For example, eating disorders can lead to the failure of the body’s cardiovascular, endocrine, gastrointestinal and neurological systems causing severe morbidity and eventual mortality [53]. The rise in psychiatric illnesses may, also, increase the risk of suicide [53]. The resulting effects of body dissatisfaction and anxiety and depression may therefore be damaging towards many different aspect of men’s health.

Financial burdens may also be associated with anxiety and depression in body dissatisfied men, as health risk behaviours such as diet pill and steroid use are often expensive [3]. And if these behaviours do become obsessive this will result in even further expenditure. Male students with body dissatisfaction have demonstrated these behaviours in an attempt to improve their muscularity and thinness [54], highlighting that these costly practices are even important to students, therefore, further reducing financial security in an already low income group.

Body dissatisfaction and anxiety and depression could, additionally, lead to social consequences. The Gender Role Conflict Theory proposes that body dissatisfied males may suffer an inner conflict between their vulnerabilities and not wanting to appear visibly weak, and instead choose to appear strong and confident [55; 56]. For example, men may avoid counselling and express dominance over others as compensatory behaviour for their perceptions of their own bodies [55]. Shepherd and Rickard [57] found that men with an increased drive for muscularity are less likely to seek help for their body dissatisfaction, which increases the risk of poor mental health. Consequently, these feelings and behaviours could worsen if associated with anxiety and depression, as they could intensify men’s vulnerability caused by body dissatisfaction, and ultimately lead to a breakdown in relationships and negativity in social settings. In summary body dissatisfaction is an important subject, and may lead to severe outcomes if associated with anxiety and depression in men.

### Limitations and strengths of systematic review

The systematic review was not conducted by a secondary reviewer, which increased the risk of researcher bias. It is recommended that a secondary reviewer is utilised to help ensure that methods are conducted reliably [58]. Siddaway *et al* [59] do concur that it is best practice to employ secondary reviewers, though, state it is also possible for a single reviewer to conduct a high quality and reputable systematic review independently due to their highly structured and organised nature. The use of validated methods employed ensured that pre-existing instructions had to be closely followed and repeated each time, meaning that the systematic review was conducted in an orderly and rigorous way. Additionally, all aspects of the systematic review were agreed by all authors prior to being completed. For these reasons the risks of poor reliability were reduced to some extent.

Other limitations included the decisions to use English and peer-reviewed literature only, which reduced the scope of literature available to inform the systematic review. However, the PRISMA flowchart confirmed that few studies were excluded because they were non-English, thereby suggesting that the risk of language bias was low.

In systematic reviews the risk of publication bias needs to be assessed. Studies reporting higher effect sizes are more often published [60], which increases publication bias. This is because systematic reviews and meta-analyses only including these published studies may conclude that a false-positive relationship exists. Statistical tests for publication bias in this review did not indicate the presence of small study bias as determined by Begg’s rank correlation test. There is a chance that there was omitted evidence in unpublished articles as this review included published studies only. Future research should, consequently, utilise larger sample sizes and include unpublished evidence sources such as reports and theses.

This review demonstrated pooled correlations of 0.40 (95% CI 0.28 to 0.51) for anxiety, 0.34 (95% CI 0.22 to 0.45) for depression, and 0.47 (95% CI 0.33 to 0.59) for combined outcomes with body dissatisfaction. This indicates that anxiety accounts for 16%, depression accounts for 12%, and combined anxiety and depression accounts for 22% of the variance in reported body dissatisfaction. These effects are not additive.

The restricted publication age to 2008–2018 has provided a valuable snapshot during the most important time in recent history that male body dissatisfaction has been acknowledged [2; 5; 12]. This restriction has, however, prevented a valuable opportunity in assessing how the relationship between male body dissatisfaction and anxiety and depression has changed over time. Future reviews should, therefore, consider how the relationship between these variables has evolved and what factors have influenced this.

The review has many strengths despite these limitations, including the use of validated methods to formulate a research aim, conduct a literature search and strategy, perform data extraction and quality appraisal, and undertake data analysis and synthesis. The studies selected to inform the systematic review were rigorously assessed against extensive eligibility criteria that ensured only relevant studies were identified, analysed and synthesised to generate findings. By employing these validated methods, the review was therefore high in quality and could be transparently reported.

## Conclusion

The systematic review and meta-analysis have confirmed that body dissatisfaction appears to be related to anxiety and depression in otherwise healthy adult males. A positive correlation between these variables was commonly reported amongst studies identified in the literature search, and in all three meta-analyses there was a statistically significant and positive relationship between male body dissatisfaction and anxiety and/or depression. In light of these findings, understanding the relationship between the mental health of men and how this could be affected by body dissatisfaction should, therefore, be the subject of further research. Longitudinal designs are necessary to understand the temporal relationships between anxiety, depression and body dissatisfaction in men, and in addition, these relationships should be assessed in a more diverse samples of men.

## Supporting information

S1 Fig PRISMA flow chart. (DOCX)

S2 Fig Meta-analysis to show the correlational relationship between body dissatisfaction and anxiety in otherwise healthy adult males. (DOCX)

S3 Fig Meta-analysis to show the correlational relationship between body dissatisfaction and depression in otherwise healthy adult males. (DOCX)

S4 Fig Meta-analysis to show the correlational relationship between body dissatisfaction and both anxiety and depression in otherwise healthy adult males. (DOCX)

S1 Table Preferred reporting items for systematic reviews and meta-analyses (PRISMA) guidelines. (DOCX)

S2 Table Search history. (DOCX)

S3 Table Data extraction worksheet. (DOCX)

S4 Table Quality appraisal findings. (DOCX)

## Abbreviations

AXIS Tool: Appraisal tool for Cross-Sectional Studies
BMI: Body Mass Index
PRISMA: Preferred Reporting Items for Systematic Reviews and Meta Analyses
SPIDER: Sample; Phenomenon of Interest; Design; Evaluation; Research Type
