# Supplementary figures and images for: Associations between body dissatisfaction and self-reported anxiety and depression in otherwise healthy men: A systematic review and meta-analysis
Source: PLoS One. 2020 Feb 25;15(2):e0229268. doi: 10.1371/journal.pone.0229268 (PMC7041842; doi:10.1371/journal.pone.0229268)

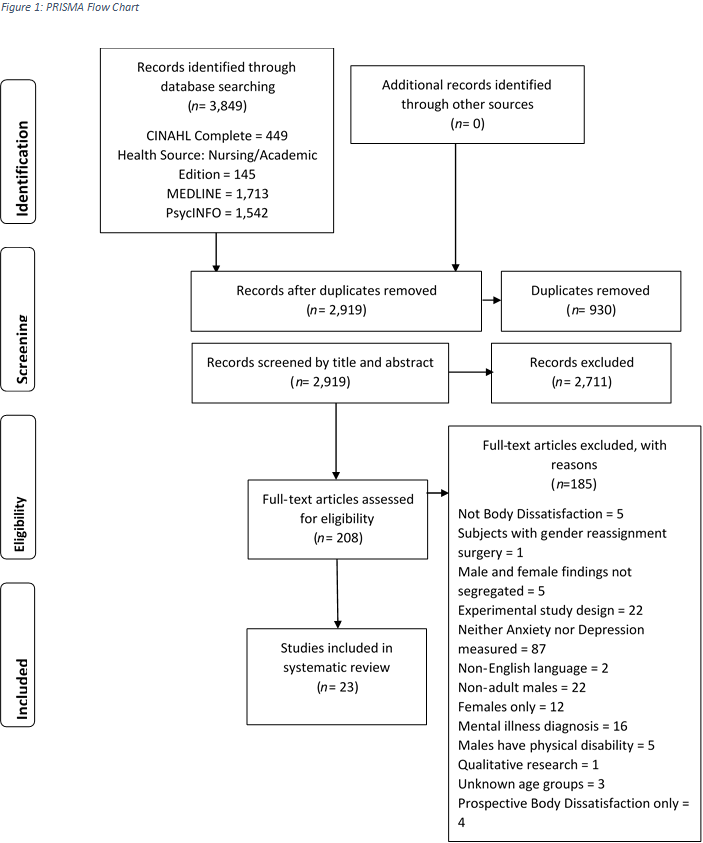


Studies included in content analysis

(*n*= 23)

Studies included in meta-analysis

(*n*= 18)

Supplement: S1 Fig — (DOCX) [file pone.0229268.s001.docx]
