# Supplementary material for: Associations between body dissatisfaction and self-reported anxiety and depression in otherwise healthy men: A systematic review and meta-analysis
Source: PLoS One. 2020 Feb 25;15(2):e0229268. doi: 10.1371/journal.pone.0229268 (PMC7041842; doi:10.1371/journal.pone.0229268)

*Figure 4: Meta-Analysis to show the correlational relationship between body dissatisfaction and both anxiety and depression in otherwise healthy adult males.*


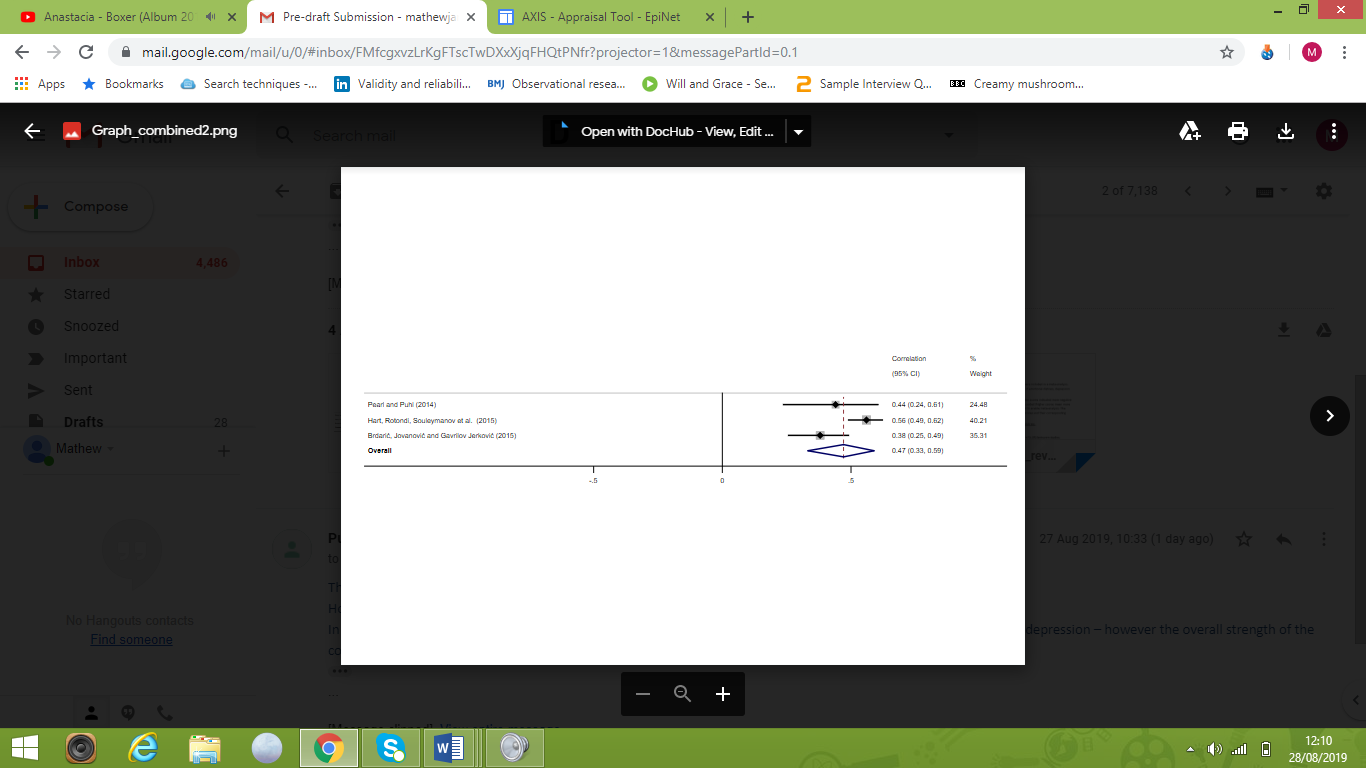

Supplement: S4 Fig — (DOCX) [file pone.0229268.s004.docx]
