# Supplementary material for: Associations between body dissatisfaction and self-reported anxiety and depression in otherwise healthy men: A systematic review and meta-analysis
Source: PLoS One. 2020 Feb 25;15(2):e0229268. doi: 10.1371/journal.pone.0229268 (PMC7041842; doi:10.1371/journal.pone.0229268)
Supplement: S1 Table — (DOCX) [file pone.0229268.s005.docx]

**Preferred Reporting Items for Systematic Reviews and Meta-Analyses (PRISMA) guidelines**

Section/topic # Page Reported

TITLE

Title 1 1

ABSTRACT

Structured summary 2 2-3

INTRODUCTION

Rationale 3 4-5

Objectives 4 5

METHODS

Protocol and registration 5 5

Eligibility criteria 6 5-7

Information sources 7 7

Search 8 7

Study selection 9 7-8

Data collection process 10 9

Data items 11 9

Risk of bias in individual studies 12 8-9

Summary measures 13 9-10

Synthesis of results 14 10-11

Risk of bias across studies 15 N/A

Additional analyses 16 N/A

RESULTS

Study selection 17 8

Study characteristics 18 11-24

Risk of bias within studies 19 24-26

Results of individual studies 20 26-29

Synthesis of results 21 30-32

Risk of bias across studies 22 N/A

Additional analysis 23 N/A

DISCUSSION

Summary of evidence 24 32-37

Limitations 25 37-39

Conclusions 26 39

FUNDING

Funding 27 N/A
