# Supplementary material for: Associations between body dissatisfaction and self-reported anxiety and depression in otherwise healthy men: A systematic review and meta-analysis
Source: PLoS One. 2020 Feb 25;15(2):e0229268. doi: 10.1371/journal.pone.0229268 (PMC7041842; doi:10.1371/journal.pone.0229268)
Supplement: S2 Table — (DOCX) [file pone.0229268.s006.docx]

**Search History**

*Database: MEDLINE Date: 10th April 2018*

| **Search No.** | **Search Undertaken** | **No. of Results** |
| --- | --- | --- |
| 1 | adult* (TI (Title), AB (Abstract)) | 1,064,792 |
| 2 | male* (TI, AB) | 992,622 |
| 3 | m?n (TI, AB) | 1,352,510 |
| 4 | “Aged, 80 and Over” (MeSH 2017) | 1,446 |
| 5 | “Middle Age” (MeSH 2017) | 543 |
| 6 | “Young Adult” (MeSH 2017) | 87 |
| 7 | 1 OR 2 OR 3 OR 4 OR 5 OR 6 | 3,053,858 |
| 8 | "body dissatisfaction" (TI, AB) | 2,097 |
| 9 | "body satisfaction" (TI, AB) | 626 |
| 10 | "body image" (TI, AB) | 8,801 |
| 11 | "body sha?e" (TI, AB) | 2,701 |
| 12 | "body sham*" (TI, AB) | 112 |
| 13 | appearance (TI, AB) | 206,272 |
| 14 | "ideal body" (TI, AB) | 2,756 |
| 15 | "desir* body" (TI, AB) | 231 |
| 16 | "body type" (TI, AB) | 1,030 |
| 17 | "body size" (TI, AB) | 17,852 |
| 18 | body *N3* dissatisfaction (TI, AB) | 2,702 |
| 19 | body *N3* satisf* (TI, AB) | 1,514 |
| 20 | body *N3* image (TI, AB) | 9,602 |
| 21 | body *N3* sha?e (TI, AB) | 4,075 |
| 22 | body *N3* sham* (TI, AB) | 559 |
| 23 | ideal *N3* body (TI, AB) | 3,218 |
| 24 | desir* *N3* body (TI, AB) | 658 |
| 25 | body *N3* type (TI, AB) | 4,336 |
| 26 | body *N3* size (TI, AB) | 21,458 |
| 27 | 8 OR 9 OR 10 OR 11 OR 12 OR 13 OR 14 OR 15 OR 16 OR 17 OR 18 OR 19 OR 20 OR 21 OR 22 OR 23 OR 24 OR 25 OR 26 | 247,040 |
| 28 | Anxi* (TI, AB) | 171,584 |
| 29 | Depress* (TI, AB) | 398,231 |
| 30 | Test Anxiety Scale (MeSH 2017) | 125 |
| 31 | 29 OR 30 OR 31 OR 32 | 493,397 |
| 32 | 7 AND 27 AND 31 | 1,713 |
