# Supplementary material for: Associations between body dissatisfaction and self-reported anxiety and depression in otherwise healthy men: A systematic review and meta-analysis
Source: PLoS One. 2020 Feb 25;15(2):e0229268. doi: 10.1371/journal.pone.0229268 (PMC7041842; doi:10.1371/journal.pone.0229268)
Supplement: S3 Table — (DOCX) [file pone.0229268.s007.docx]

**Data Extraction Worksheet**

| **Study ID** |  |
| --- | --- |
| **Author, Year** |  |
| **Aims/Objectives** |  |
| **Country** |  |
| **Setting** |  |
| **Body Dissatisfaction type (body tan, genital self-image, muscularity and thinness, or adiposity and weight)** |  |
| **Health Outcomes assessed (must include either anxiety or depression)** |  |
| **Adult Male Population under investigation** |  |
| **Male Sample Size (n= and % of total sample)** |  |
| **Ages** |  |
| **Ethnicity (% per group)** |  |
| **Education Status (% per group)** |  |
| **Sexuality (% per group)** |  |
| **Positive Association between Body Dissatisfaction and Anxiety?** |  |
| **Positive Association between Body Dissatisfaction and Depression?** |  |
| **Quality Appraisal completed? Have all sections been answered?** |  |
