# Supplementary material for: Associations between body dissatisfaction and self-reported anxiety and depression in otherwise healthy men: A systematic review and meta-analysis
Source: PLoS One. 2020 Feb 25;15(2):e0229268. doi: 10.1371/journal.pone.0229268 (PMC7041842; doi:10.1371/journal.pone.0229268)
Supplement: S4 Table — (DOCX) [file pone.0229268.s008.docx]

**Quality Appraisal Findings**

**Key**

Y = Yes
N = No/Not Reported

|  | Introduction | Methods | | | | | | | | | | Results | | | | | Discussion | | Other | |
| --- | --- | --- | --- | --- | --- | --- | --- | --- | --- | --- | --- | --- | --- | --- | --- | --- | --- | --- | --- | --- |
| Author, Year | Clear Aims/ Objectives | Appropriate Study Design | Justified Sample Size | Defined Target Population | Appropriate Sampling Frame | Appropriate Sampling Method | Non-Respondents Addressed | Risk and Outcome Variables Appropriate | Valid Data Collection Tools Used | Use of Statistical Significance and Precision Estimates | Study is Repeatable | Adequate Data Description | Non-Response Bias Concerns | Description of Non-Respondents | Internal Consistency of Results | All Analyses Described | Conclusions Justified by Results | Limitations Reported | Funding/ Conflicts of Interest Declared | Ethics/Consent in place |
| [24] | Y | Y | N | N | Y | N | N | Y | Y | N | N | Y | Y | N | N | Y | Y | Y | N | Y |
| [25] | Y | Y | N | N | Y | N | N | Y | Y | Y | N | Y | Y | N | N | Y | Y | Y | N | Y |
| [26] | Y | Y | N | N | Y | Y | N | Y | Y | N | N | Y | N | N | Y | Y | Y | Y | N | Y |
| [27] | Y | Y | N | N | N | N | N | Y | Y | N | N | Y | Y | N | Y | Y | Y | Y | N | Y |
| [28] | Y | Y | N | N | Y | N | N | Y | Y | N | N | Y | N | N | Y | Y | Y | Y | N | Y |
| [29] | Y | Y | N | N | N | N | N | Y | Y | N | N | Y | Y | N | Y | Y | Y | Y | N | Y |
| [30] | Y | Y | N | N | N | N | N | Y | Y | N | N | Y | Y | N | Y | Y | Y | Y | N | Y |
| [31] | Y | Y | N | N | N | N | N | Y | Y | Y | N | Y | Y | N | Y | Y | Y | Y | N | Y |
| [32] | Y | Y | N | N | Y | N | N | Y | Y | Y | N | Y | Y | N | Y | Y | Y | N | N | Y |
| [33] | Y | Y | Y | Y | N | N | N | Y | Y | Y | N | Y | Y | N | Y | Y | Y | Y | N | Y |
| [34] | Y | Y | N | Y | Y | N | N | Y | Y | N | N | Y | Y | N | Y | Y | Y | Y | N | Y |
| [35] | Y | Y | N | N | Y | N | N | Y | Y | N | N | Y | Y | N | Y | Y | Y | Y | N | Y |
| [36] | Y | Y | N | N | N | N | N | Y | Y | N | N | Y | Y | N | N | Y | Y | Y | N | N |
| [37] | Y | Y | N | N | N | N | N | Y | Y | N | N | Y | Y | N | Y | Y | Y | Y | N | Y |
| [38] | Y | Y | N | N | N | N | N | Y | Y | N | N | Y | Y | N | N | Y | Y | Y | N | N |
| [39] | Y | Y | N | N | Y | Y | N | Y | Y | Y | Y | Y | Y | N | Y | Y | Y | Y | N | Y |
| [40] | Y | Y | N | N | Y | Y | Y | Y | Y | Y | Y | Y | N | N | Y | Y | Y | Y | N | Y |
| [41] | Y | Y | N | N | N | N | N | Y | Y | N | N | Y | Y | N | N | Y | Y | Y | N | Y |
| [42] | Y | Y | Y | Y | N | N | N | Y | Y | N | N | Y | Y | N | Y | Y | Y | Y | N | Y |
| [43] | Y | Y | N | N | N | N | N | Y | Y | Y | N | Y | Y | N | Y | Y | Y | Y | N | Y |
| [44] | Y | Y | N | N | N | N | N | Y | Y | N | N | Y | Y | N | Y | Y | Y | Y | Y | Y |
| [45] | Y | Y | N | N | N | N | N | Y | Y | Y | N | Y | N | N | Y | Y | Y | Y | N | Y |
| [46] | Y | Y | N | N | N | N | N | Y | Y | N | N | Y | N | N | N | Y | Y | Y | N | Y |
